# Assessing the second-hand effects of a new no-smoking policy in an acute mental health trust

**DOI:** 10.1192/pb.bp.116.055749

**Published:** 2017-12

**Authors:** Benjamin Ian Perry, Katherine Meehan, Ashok Kumar Jainer

**Affiliations:** 1 University of Warwick; 2 Coventry and Warwickshire Partnership NHS Trust

## Abstract

**Aims and method** To examine whether a new no-smoking policy in an in-patient mental health setting had any effects outside of smoking cessation. Our hypothesis stated that a forced smoking ban for in-patients may result in an increased susceptibility for clinical incidents, aggression and lower admission rates. All patients admitted to adult in-patient mental health services in Coventry and Warwickshire Partnership NHS Trust were included in the analysis. Data 6 months post-implementation of the smoking policy (1 July 2015 to 1 January 2016) were compared with the same period 1 year prior (1 July 2014 to 1 January 2015). Patient demographics, admission rates, ward occupancy, average lengths of stay, numbers of reported incidents and use of the Mental Health Act 1983 (MHA) were compared.

**Results** We analysed 4223 admissions. We found a significantly increased number of admissions under the MHA (*P* = 0.007), a significantly greater number of reported smoking-related incidents (*P* < 0.001) and aggression-related incidents in the psychiatric intensive care unit (*P* < 0.001). However, we found no significant difference in capacity of in-patient wards (*P* = 0.39), admission length (*P* = 0.34) or total aggression-related incidents (*P* = 0.86).

**Clinical implications** Although further comparisons over longer time periods are necessary, our results suggest that enforced smoking cessation on acutely unwell psychiatric patients admitted to the most restricted environments may have some negative effects. Nicotine replacement therapy should be offered to all patients to minimise the risk of clinical incident.

Smoking rates among those with a mental illness are 70% higher than in the general population.^1^ In fact, although the prevalence of smoking in the general population has decreased, this has not occurred for those with a mental illness.^2^ In particular, the highest smoking rates are found in those acutely unwell with a psychiatric illness, in in-patient units.^3–6^

Multiple explanations for such high smoking rates within the mental health sector have been put forward, including symptom control and amelioration,^7^ boredom or loneliness,^8,9^ an increased propensity to experience more severe nicotine withdrawal symptoms than the general population,^10,11^ for relaxation in a stressful environment,^9,12^ a common genetic vulnerability^5^ or that people with a mental illness are less susceptible to anti-smoking messages.^13^

As a result of relatively higher smoking rates, people with a mental illness also have higher mortality rates than the general population. Tobacco use contributes significantly to causes of ill health and mortality in those with mental health disorders.^11^ Individuals at particular risk are patients with schizophrenia, who have a life expectancy 20% shorter than the general population.^14,15^ Tobacco use can also affect the effectiveness of some psychiatric medication, necessitating increased dosages and therefore purporting a higher chance of side-effects.^16^

The UK government implemented its smoke-free policy in July 2007, which extended to all ‘substantially enclosed’ public and work places. This included hospitals, with the aim of reducing the impact of second-hand smoke on patients and staff^17^ This was extended to all types of in-patient units from 2008.

Guidance from the National Institute for Health and Care Excellence (NICE), published in 2013, aims to support smoking cessation, temporary abstinence from smoking and smoke-free policies in all secondary care settings.^18^ In this guidance, ‘secondary care’ refers to all publicly funded secondary and tertiary care facilities, including buildings, grounds and vehicles. It covers in-patient, residential and long-term care for severe mental illness in hospitals, psychiatric settings, specialist and secure units.

Prior to the introduction of the legislation, a relatively large survey of National Health Service (NHS) staff found that a third of psychiatric staff disagreed with smoke-free legislation compared with only one in ten of general staff.^19^ A survey of mental health units in England in January 2007 found that the vast majority (91%) believed mental health premises faced particular challenges due to the high smoking prevalence among patients, associated safety risks, and potential interactions with antipsychotic medication.^20^ However, despite the challenges, the smoke-free policy has been rated positive overall. Cited advantages include a reduced exposure of patients and staff to second-hand smoke, an enhancement and support in patients' motivation to stop smoking, improved sleeping patterns among patients, and the conversion of former smoking rooms into new recreational spaces.^20^

Since the development of non-smoking policies within the NHS, debate has evolved around any potential infringement this might have on a patient's human rights. A patient at one of Scotland's high-security forensic hospitals recently won a court ruling that a blanket ban on smoking breached his human rights.^21^ According to Article 8 of the Human Rights Act 1998, everyone has the right to respect for his private life and his home. With regard to mental health units, lengthy hospital admissions may qualify as breach of one's private and home life.

The introduction of a non-smoking policy to mental health services remains a relatively novel practice. We are yet to fully appreciate its impact on in-patient services. Coventry and Warwickshire Partnership NHS Trust in the UK introduced a no-smoking policy within its mental health units on 1 July 2015. The Trust is one of the first to implement this policy following the growing evidence of high smoking rates and adverse health implications within the mental health population. Although the benefits of smoking cessation are widely acknowledged, there exists an assumption that enforcing smoking cessation on unwilling patients results in increased stress levels and therefore higher rates of aggression-related incidents.

Voluntary admission to an in-patient mental health ward requires the patient to agree to certain ward policies and procedures explained by the clinician. Within the Trust it is standard procedure to explain the non-smoking policy for both voluntary and involuntary admissions. The impacts outside of smoking cessation caused by a forced no-smoking policy has previously been analysed in a medium secure unit, finding no significant difficulties and that the widely anticipated problems did not materialise.^22^ We have been unable to find another study analysing similar changes in a typical psychiatric unit (mixed voluntary and involuntary patients).

## Aims and objectives

The aim of the study was to examine whether a newly implemented no-smoking policy for patients in a typical in-patient mental health setting had any effects outside of smoking cessation. The objectives were first to compare admission rates and bed occupancy/capacity levels at comparable time periods pre- and post-implementation of the new no-smoking policy in a specified mental health trust. We also sought to compare the numbers of reported incidents occurring on the wards at comparable time periods pre- and post-implementation, focusing particularly on aggression-related and smoking-related incidents. In addition, we wanted to ascertain whether there was any significant difference in the use of the Mental Health Act 1983 at comparable time periods pre- and post-implementation of the new policy, and whether this was related to the change in smoking policy.

## Method

### Study location and Trust smoking policy

Data were collected from all patients admitted to mental health beds in Coventry and Warwickshire Partnership NHS Trust during the 12 months before and 6 months after implementation of the smoking ban. The change in policy was implemented on 1 July 2015, therefore data were collected between 1 July 2014 and 1 January 2016. The Trust smoking ban states that ‘all staff, patients and visitors are not able to smoke tobacco products in Trust buildings or on Trust land.’^23^ The Trust maintains a policy of offering nicotine replacement therapy to admitted patients, comprising of either an e-cigarette or nicotine transdermal patch.

Adult in-patient mental health services in the Trust comprise of three acute psychiatric units: the Caludon Centre in Coventry (112 beds), St Michael's Hospital in Warwick (41 beds) and the Pembleton Unit in Nuneaton (12 beds), with adult rehabilitation services provided at multiple sites (40 beds), for a catchment area of around 850 000 people.

### Inclusion and exclusion criteria

All patients admitted to adult in-patient mental health services, both acute and rehabilitation, in Coventry and Warwickshire Partnership NHS Trust were included in analysis. To account for seasonal variation, data 6 months post-implementation of the smoking policy (1 July 2015 to 1 January 2016) were compared with the same 6 months the year prior to implementation of the smoking policy (1 July 2014 to 1 January 2015). There were no specific inclusion criteria for diagnosis or length of admission to help prevent selection bias. All sites within the Trust were included in the analysis.

### Ethics

The study was approved by Coventry and Warwickshire Partnership NHS Trust as a service evaluation and as such did not need formal ethical approval from an NHS research ethics committee. Data were collated in an anonymised format from routine clinical records, by the authors.

### Data collection

First, basic demographic data such as mean age and gender were obtained. Second, monthly admission rates and ward occupancy levels between the dates were collected. Third, monthly total numbers of reported incidents were obtained. All data were collected by data analysts within the Trust.

### Statistical analysis

For the count data (number of admissions under the MELA, total incidents, aggression-related incidents, psychiatric intensive care unit (PICU) incidents and smoking incidents), Poisson regression was used to generate a significance value. Where data were provided as percentages (i.e. capacity), we converted to mean *n* based on the total Trust capacity (*n* = 205). The Shapiro-Wilk test for normality allowed a decision as to whether to use parametric or non-parametric statistical comparisons. All statistical comparisons were made using IBM SPSS Statistics 24.

Since we were are measuring six outcomes in our analysis, a Bonferroni correction was applied. The α-value (0.05) was therefore adjusted to a significance value of *P* = 0.008.

## Results

### Demographic data

Table 1 outlines the demographic data comparisons during our two selected periods of analysis. Table 2 outlines the findings from our other objectives.

**Table 1:** Demographic differences

	Pre-implementation	Post-implementation
*n*	2124	2099

Male, %	60.2	59.9

Age, years: mean	29.56	29.39

**Table 2:** Data outlining differences before/after implementation of no-smoking policy

	Jul	Aug	Sep	Oct	Nov	Dec	Mean
Number of admissions under Mental Health Act 1983, *n*							
Before	143	144	145	171	169	173	157.5
After	207	184	141	174	188	169	177.2

In-patient ward capacity, %							
Before	101	102	100	101	104	104	102.0
After	104	101	102	104	98	101	101.7

In-patient ward capacity, mean *n*							
Before	207.5	209.1	205	207.5	213.2	213.2	209.3
After	217.2	207.5	209.1	213.2	200.9	207.5	209.2

Mean duration of stay, days							
Before	38.1	38.8	40.6	44.3	55.7	36.6	42.4
After	44.7	37.0	37.2	37.8	41.9	36.3	45.4

Total aggression-related incidents, *n*							
Before	105	87	59	48	82	88	78.2
After	76	92	51	93	79	90	80.2

Aggression-related incidents on PICU, *n*							
Before	24	16	16	22	21	13	18.6
After	32	20	35	25	37	29	29.6

Smoking-related incidents, *n*							
Before	9	7	9	7	2	5	6.5
After	38	19	17	12	9	26	20.2

PICU, psychiatric intensive care unit.

### Number of patients admitted under the Mental Health Act 1983

Poisson regression found that the number of admissions under the MHA increased (1.13, 95% CI 1.03–1.23) at the boundary of our corrected α-value, *P* = 0.007, in the same 6 months the year following the introduction of the new smoking policy.

### In-patient ward capacity

Our findings show that the bed capacity was at maximum or over-maximum at each month studied. Using the data adjusted into mean capacity, the Shapiro-Wilk test for normality (*P* = 0.306) allowed us to proceed with an unpaired *t*-test, which showed no significant difference (*P* = 0.99).

### Average duration of in-patient admission in days

The Shapiro-Wilk test for normality (*P* = 0.068) allowed us to proceed with an unpaired *t*-test, which showed no significant difference (*P* = 0.34).

### Total number of in-patient aggression-related incidents

Poisson regression revealed no significant difference in total aggression-related incidents following the introduction of the new smoking policy (1.02, 95% CI 0.90–1.12; *P* = 0.70).

### Aggression-related incidents (PICU only)

Poisson regression revealed a significant increase in aggression-related incidents in PICU following the introduction of the new smoking policy (1.59, 95% CI 1.26–2.01; *P* < 0.001).

### Smoking-related incidents

Poisson regression revealed a significant increase in smoking-related incidents following the introduction of the new smoking policy (3.10, 95% CI 2.55–4.46; *P* < 0.001).

## Discussion

### Main findings

We aimed to ascertain whether a new no-smoking policy for in-patients at a specified mental health trust might result in any less favourable effects outside of smoking cessation. We found a statistically significant increase in the number of admissions under the MHA, total number of reported aggression-related incidents on PICU, and a statistically significant increase in the number of reported smoking-related incidents. The majority of these findings may be expected. In consideration with our finding that in-patient bed capacity was at or over 100% for each of the months studied (potentially due to patients being ‘on leave’ from hospital but still named in beds), one might argue that the acutely stressed state necessary to be granted an admission into bedspace at a premium would have been of considerable severity. Such patients may also have been admitted against their will, further heightening stress levels. One may therefore consider that immediately and forcefully removing the right to smoking, a past-time that can bring comfort, reduce stress, ameliorate psychiatric symptoms and help to fight boredom, may be poorly timed.

Perhaps a more surprising finding is the statistically significant increase in patients admitted under the MHA This was included as an outcome measure as it was hypothesised that patients may refuse informal admission based on the no-smoking policy. Although patient refusal for informal admission may have contributed to the effect, it is likely not the whole story, as that hypothesis relies on the provision of adequate information to patients, i.e. the new smoking policy is explained prior to admission. Other work^24^ has shown that this is not always the case. It is also well known that detentions under the MHA have been on the rise across the UK over the past 10 years,^25^ due to a multitude of factors (not smoking related) which we were unable to analyse in this study. In-depth case-note analysis may have allowed us to qualitatively ascertain whether the smoking policy played a part in this significant finding, and future research analysing this perhaps legitimate question could take this into account.

We found no significant difference in the total number of reported aggression-related incidents. At face value, this finding suggests that in an open-ward environment, the new smoking policy did not cause an increase in agitation or aggression, which contradicts the finding we obtained from PICU only There are several possible explanations for this. First, the patients that are admitted to PICU are likely to be more acutely stressed than those admitted to an open ward and therefore the potential to cause an ‘incident’ might be increased. Second, the more strictly controlled environment in PICU may lend itself to better adherence of the smoking policy than for informal patients on an open ward (who may be allowed out for ‘grounds leave’ each hour, or more), thus the new smoking policy may be felt more among patients on PICU Third, there is the very likely possibility that not all incidents are reported. Incident reporting can sometimes be viewed as an arduous process, especially for staff with busy in-patient ward roles. Although this could affect the results both in the open-ward environment and on PICU, one could suggest that staff on PICU might be more familiar with and better trained to deal with incidents, thus incident reporting might be better adhered to.

We also found no significant difference in patients' length of admission, suggesting that the new no-smoking policy did not positively or negatively affect the patient journey through mental health services. This might be an expected finding as the benefits of smoking cessation are known for long-term rather than short-term health. However, it is useful to address this result in light of our findings of increased smoking-related and aggression-related incidents, as it suggests that the new policy's potential to predispose to aggression or agitation does not necessarily result in prolonged in-patient stay. We also found no significant difference in in-patient ward capacity, which could be expected considering capacity was at maximum or above maximum for each month studied.

### Strategies and limitations

We believe this study is one of the first to assess the effects of a new no-smoking policy of psychiatric in-patients in the UK, in an age where the importance of physical health in psychiatric patients is becoming increasingly recognised, such that many more healthcare trusts may in future choose to adopt a similar policy. In using the entire sampling frame over a 6-month period, we have ensured a large sample size which may help to reduce the potential for type I or II statistical errors. We have reduced the impact of seasonal variation by comparing the same 6 months both in the year of introduction of the new no-smoking policy and the year preceding it. In comparing data across time, we can demonstrate a temporal association with the positive findings. Furthermore, regarding the new no-smoking policy being more strictly enforced on PICU, a dose-response relationship may be observed when comparing the non-significant open ward aggression-related findings with those obtained from PICU In addition, we have included a range of measures that were chosen prior to commencing data collection.

There are however a number of limiting factors that should be taken into consideration. First and most importantly, we cannot show that the new no-smoking policy is causal to the positive findings. There could be many other causes for increased aggression-related incidents on PICU and it is therefore not clear how much (if at all) the change in smoking policy contributed. It is however less probable to consider reverse causality as a factor in this study, as it is unlikely that the no-smoking policy was enacted because of significant aggression-related incidents.

In addition, we are unable to determine the effect of any poor reporting practice on our findings. We have mentioned that reporting might be better in certain areas of in-patient psychiatric care than others which may skew our results and invalidate comparisons. Despite our efforts, we were unable to obtain a comparison of total number of reported incidents between the two time periods. This would have better highlighted the reporting practices in the Trust across the two time periods and may be useful for future work.

It may also be possible that the timing of our data collection may have affected the validity of our results. We chose to measure the 6 months immediately following the introduction of the new no-smoking policy. It is likely that group practice takes time to adapt and this may be an explanation for some of the negative findings in our results. It may have been more appropriate to choose a length of inclusion greater than 6 months to better visualise this, however we were limited by time and resources. Both adherence to the Trust smoking policy and the reporting of incidents (if common) may improve over time, and it therefore may be useful to repeat this study in the future to compare the results.

Finally, since our study only measured outcomes during in-patient stay, we were unable to ascertain any longer-term effects of the new smoking policy, such as the increased achievement of smoking cessation among patients admitted under the new no-smoking policy. Further work might seek to establish the longer-term effects of such a policy.

Other research has been carried out on this topic internationally, which correlates partially with our results. A 2002 systematic review^26^ including studies from several countries found no significant behavioural effects when smoking bans were enacted in psychiatric units, although the review also notes that in the included studies, smoking bans were not associated with long-term smoking cessation among patients. More recently, a 2005 study^27^ from the USA found no significant increase in aggression with the introduction of a new smoking ban. Furthermore, a survey^28^ of mental health staff working at an Australian healthcare trust in 2013 found that although most staff preferred to work in a smoke-free environment, around half of survey respondents found the smoking ban to be detrimental to acute patient care, which may give the impression of increased agitation or behavioural problems among patients involved.

Our results are therefore broadly in line with the findings of others. However, it is notable that we have shown a potential difference in the effects of a smoking ban on different patient groups across different settings. Further work might seek to clarify and further examine the reasons behind this finding.

Overall, the reasoning behind a smoke-free hospital environment is clear. The long-term health benefits of smoking cessation are numerous and well documented, and other work has shown staff to prefer a smoke-free working environment. However, there is the legitimate debate as to whether the acutely stressed state is the right time to impose this lifestyle change, and whether it is even ethical to do so. Our results show that, in general, a new smoke-free policy did not result in significant changes of reported aggressive behaviour or incidents, and did not affect the patient journey through mental health services. However, we found significantly increased smoking- and aggression-related incidents in more restricted environments. It is possible that the most highly agitated psychiatric patients are most susceptible to cause incidents with this enforced lifestyle change. Our finding of significantly increased use of the MHA may be explained by other factors, but also may warrant further research. Therefore, to reduce the impact this may have on both patients and staff tasked with dealing with any resultant incident, nicotine replacement therapy should be considered for all relevant patients.

## References

[1] Centers for Disease Control and Prevention Smoking among U.S. adults with mental illness 70 percent higher than for adults with no mental illness. CDC, 2012.

[2] Szatkowski L McNeill A Diverging trends in smoking behaviors according to mental health status. Nicotine Tob Res 2015; 17: 356–60. 25180078 10.1093/ntr/ntu173 PMC5479511

[3] Meltzer H Economic Activity and Social Functioning of Residents with Psychiatric Disorders: p. 83 HMSO, 1996.

[4] Meltzer H Gill B Hinds K Petticrew M The prevalence of psychiatric morbidity among adults living in institutions. Int Rev Psychiatry 2003; 15: 129–33. 12745320 10.1080/0954026021000046047

[5] Royal College of Psychiatrists Smoking and mental health. Royal College of Psychiatrists, 2017.

[6] Jochelson K Majrowski B Clearing the Air: Debating Smoke-Free Policies in Psychiatric Units. King's Fund, 2006.

[7] Smith RC Singh A Infante M Khandat A Kloos A Effects of cigarette smoking and nicotine nasal spray on psychiatric symptoms and cognition in schizophrenia. Neuropsychopharmacology 2002; 27: 479–97. 12225705 10.1016/S0893-133X(02)00324-X

[8] Lasser K Boyd JW Woolhandler S Himmelstein DU McCormick D Bor DH Smoking and mental illness: a population-based prevalence study. JAMA 2000; 284: 2606–10. 11086367 10.1001/jama.284.20.2606

[9] Ratschen E Britton J McNeill A Implementation of smoke-free policies in mental health in-patient settings in England. Br J Psychiatry 2009; 194: 547–51. 19478296 10.1192/bjp.bp.108.051052

[10] West R The multiple facets of cigarette addiction and what they mean for encouraging and helping smokers to stop. COPD 2009; 6: 277–83. 19811387 10.1080/15412550903049181

[11] Lawn S Campion J Factors associated with success of smoke-free initiatives in Australian psychiatric in-patient units. Psychiatr Serv 2010; 61: 300–5. 20194408 10.1176/ps.2010.61.3.300

[12] Prochaska JJ Smoking and mental illness-breaking the link. N Engl J Med 2011; 365: 196–8. 21774707 10.1056/NEJMp1105248 PMC4457781

[13] Lawrence D Mitrou F Zubrick SR Smoking and mental illness: results from population surveys in Australia and the United States. BMC Public Health 2009; 9: 285. 19664203 10.1186/1471-2458-9-285 PMC2734850

[14] Hennekens CH Hennekens AR Hollar D Casey DE Schizophrenia and increased risks of cardiovascular disease. Am Heart J 2005; 150: 1115–21. 16338246 10.1016/j.ahj.2005.02.007

[15] Joukamaa M Heliovaara M Knekt P Aromaa A Raitasalo R Lehtinen V Mental disorders and cause-specific mortality. Br J Psychiatry 2001; 179: 498–502. 11731351 10.1192/bjp.179.6.498

[16] Doll R Peto R Mortality in relation to smoking: 20 years' observations on male British doctors. BMJ 1976; 2: 1525–36. 1009386 10.1136/bmj.2.6051.1525 PMC1690096

[17] Department of Health Policy Paper. 2010 to 2015 Government Policy: Smoking. Department of Health, 2015.

[18] National Institute for Health and Care Excellence Smoking: Acute, Maternity and Mental Health Services. NICE, 2013.

[19] McNally L Oyefeso A Annan J Perryman K Bloor R Freeman S A survey of staff attitudes to smoking-related policy and intervention in psychiatric and general health care settings. J Public Health (Oxf) 2006; 28: 192–6. 16809790 10.1093/pubmed/fdl029

[20] Ratschen E Britton J McNeill A The smoking culture in psychiatry: time for change. Br J Psychiatry 2011; 198: 6–7. 21200069 10.1192/bjp.bp.110.081372

[21] Cramb A Smoking ban at state hospital breached a patient's human rights, says judge. The Telegraph 2013; 27 8.

[22] Shetty A Alex R Bloye D The experience of a smoke-free policy in a medium secure hospital. Psychiatrist 2010; 34: 287–9.

[23] Coventry and Warwickshire Partnership NHS Trust NHS trust goes ‘smokefree’ from Wednesday 1 July 2015. Coventry and Warwickshire Partnership NHS Trust, 29 6 2015.

[24] Perry B Singh SP White D Capacity assessment and information provision for voluntary psychiatric patients: a service evaluation in a UK NHS Trust. Int J Ment Health Capacity Law 2016; 22: 107–18.

[25] Care Quality Commission Monitoring the Mental Health Act in 2013/14. CQC, 2015.

[26] El-Guebaly N Cathcart J Currie S Brown D Gloster S Public health and therapeutic aspects of smoking bans in mental health and addiction settings. Psychiatr Serv 2002; 53: 1617–22. 12461225 10.1176/appi.ps.53.12.1617

[27] Matthews LS Diaz B Bird P Cook A Stephenson AE Kraus JE Implementing a smoking ban in an acute psychiatric admissions unit. J Psychosoc Nurs Ment Health Serv 2005; 43: 33–6. 16350913 10.3928/02793695-20051101-12

[28] Hehir AM Indig D Prosser S Archer VA Implementation of a smoke-free policy in a high secure mental health in-patient facility: staff survey to describe experience and attitudes. BMC Public Health 2013; 13: 315. 23566256 10.1186/1471-2458-13-315 PMC3648483

